# The Role of the Regulator Fur in Gene Regulation and Virulence of *Riemerella anatipestifer* Assessed Using an Unmarked Gene Deletion System

**DOI:** 10.3389/fcimb.2017.00382

**Published:** 2017-08-25

**Authors:** Yunqing Guo, Di Hu, Jie Guo, Xiaowen Li, Jinyue Guo, Xiliang Wang, Yuncai Xiao, Hui Jin, Mei Liu, Zili Li, Dingren Bi, Zutao Zhou

**Affiliations:** ^1^College of Veterinary Medicine, Huazhong Agricultural University Wuhan, China; ^2^Key Lab of Preventive Veterinary Medicine of Hubei Province, Huazhong Agricultural University Wuhan, China; ^3^State Key Laboratory of Agricultural Microbiology, Huazhong Agricultural University Wuhan, China

**Keywords:** *Riemerella anatipestifer*, *fur*, *pheS*, unmarked gene deletion system, virulence, Fur-box, RNA-seq

## Abstract

*Riemerella anatipestifer*, an avian pathogen, has resulted in enormous economic losses to the duck industry globally. Notwithstanding, little is known regarding the physiological, pathogenic and virulence mechanisms of *Riemerella anatipestifer* (RA) infection. However, the role of Ferric uptake regulator (Fur) in the virulence of *R. anatipestifer* has not, to date, been demonstrated. Using a genetic approach, unmarked gene deletion system, we evaluated the function of *fur* gene in the virulence of *R. anatipestifer*. For this purpose, we constructed a suicide vector containing *pheS* as a counter selectable marker for unmarked deletion of *fur* gene to investigate its role in the virulence. After successful transformation of the newly constructed vector, a mutant strain was characterized for genes regulated by iron and Fur using RNA-sequencing and a comparison was made between wild type and mutant strains in both iron restricted and enriched conditions. RNA-seq analysis of the mutant strain in a restricted iron environment showed the downregulation and upregulation of genes which were involved in either important metabolic pathways, transport processes, growth or cell membrane synthesis. Electrophoretic mobility shift assay was performed to identify the putative sequences recognized by Fur. The putative Fur-box sequence was 5′-GATAATGATAATCATTATC-3′. Lastly, the median lethal dose and histopathological investigations of animal tissues also illustrated mild pathological lesions produced by the mutant strain as compared to the wild type RA strain, hence showing declined virulence. Conclusively, an unmarked gene deletion system was successfully developed for RA and the role of the *fur* gene in virulence was explored comprehensively.

## Introduction

*Riemerella anatipestifer* (*R. anatipestifer*, RA) is non-spore forming, non-motile, Gram-negative, rod-shaped bacterium belonging to the family *Flavobacteriaceae*. Other than ducks, RA can also affect the majority of poultry including turkeys, geese, which has resulted in significant economic losses to the poultry industry worldwide. Infection leads to polyserositis and septicaemia often with neurological symptoms. At least 21 serotypes have been identified in different countries, with serotypes 1, 2, and 10 most prevalent in China (Loh et al., 1992; Cheng et al., 2003). Due to extensive genomic divergences, even within a given serotype, there is often limited cross protection and variation of virulence (Higgins et al., 2000). Presently, little is known regarding RA pathogenesis, although a number of attempts have been made to explore the molecular mechanisms underlying virulence. In previous studies, the role of outer membrane protein A (OmpA), TonB dependent receptor 1 (TbdR1), TonB family protein (TbfA), siderophore interacting protein (Sip) and CAMP cohemolysin have all been proposed as virulence associated factors (Crasta et al., 2002; Hu et al., 2011; Lu et al., 2013; Tu et al., 2014; Liu et al., 2016). All of these studies were based on gene knockout, which result in modified expression of downstream genes, known as the polar effect. As a genetic analysis tool, unmarked gene deletion system is advantageous over gene knockout strategy, as well as able to provide a more accurate estimation of gene expression and has a limited polar effect. Indeed, in many species of bacteria, such as *Enterococcus faecalis, Burkholderia* family, *Streptococcus mutans, Bacillus amyloliquefaciens*, unmarked gene deletion system has been established to elucidate molecular mechanisms of pathogenesis and virulence (Kristich et al., 2007; Barrett et al., 2008; Xie et al., 2011; Zhou et al., 2016). To the best of our knowledge, no data is available on unmarked gene deletion system in RA. The development and application of such strategies will accelerate our understanding of the mechanism of pathogenesis, virulence and antibiotic resistance in RA.

Earlier studies have established the role of Ferric uptake regulator (Fur) proteins in virulence in a variety of bacterial species (Ernst et al., 2005a; Haraszthy et al., 2006; Yuhara et al., 2008; Porcheron and Dozois, 2015; Pi et al., 2016). Fur is a regulator of transcription in bacteria, involved in iron homeostasis, acid resistance, oxidative stress and virulence (Bijlsma et al., 2002; Ernst et al., 2005b; Mathieu et al., 2016). Iron is an essential element in various metabolic pathways of bacteria and eukaryotic host (Holmes et al., 2005). To date, the function of *fur* gene in virulence of RA has not been demonstrated in any previous study. In this novel study, the role of *fur* gene in virulence of RA has been examined by adopting unmarked gene deletion system. Having observed the limitations of other counter-selectable markers, *pheS* is an appropriate non-antibiotic resistance counter-selectable marker. Previously, the applications of the mutant *E. coli pheS* gene (A294G), the mutant *E. faecalis pheS* gene (A312G), the mutant *Burkholderia pheS* gene (A294G), and the mutant *S. mutans pheS* gene (A314G) were successful for allelic replacement in those organisms (Kast and Hennecke, 1991; Ibba et al., 1994; Kristich et al., 2007; Barrett et al., 2008; Xie et al., 2011). Therefore, we postulated the role of *pheS* gene for this purpose.

In summary, in this study, we engineered a suicide vector pRE-lacZ-mpheS-spc, using mutated *pheS* as a counter-selectable marker and *lacZ* to select a *fur* gene deletion mutant RA-YM Δ*fur*. This is the first successful attempt to construct mutant RA using an unmarked gene deletion system. The RA-YM Δ*fur* complemented strain was constructed to confirm virulence of the wild type strain, compared with the mutant. Lastly, using whole genome transcriptional sequencing, genes regulated by the *fur* gene were screened out in mutant and wild types. Moreover, the predictive sequence of Fur-box of RA was analyzed.

## Materials and methods

### Bacterial strains, plasmids, media, and growth conditions

The bacterial strains and plasmids used in this study, and their relevant characteristics are described in Table 1. *R. anatipestifer* strains were grown at 37°C in tryptic soy broth (TSB) (Difco, Detroit, USA) in an atmosphere of 5% CO_2_, *E. coli* strains were cultured at 37°C in Luria Bertani broth (Sigma-Aldrich, St. Louis, USA). Both *R. anatipestifer* strains and *E. coli* strains included in this study were obtained from laboratory stocks of the Department of Veterinary Microbiology and Immunology of Huazhong Agricultural University, China. Where necessary, the following antibiotics were added in to the selection media: ampicillin (Amp), 100 mg/mL; spectinomycin (Spc), 100 mg/mL; kanamycin (Kan), 100 mg/mL; and medium was supplemented with 2, 6-diaminopimelic acid (DAP), 100 mg/mL; 5-bromo-4-chloro-3-indolyl β-D-galactopyranoside (X-gal), 20 mg/mL; Isopropylβ-D-1-thiogalactopyranoside (IPTG), 20 mg/mL; 4-chloro-DL-phenylalanine (cPhe), 0.2% (w/v).

**Table 1:** Strains, plasmids and primers.

**Strains or plasmids**	**Description**	**PCR product**	**Reference**
**STRAINS**			
RA-YM	*Riemerella anatipestifer* wild-type strain, serotype 1		This study
RA-JX	*Riemerella anatipestifer* strain, serotype 1		
RAYM Δ*fur*	*fur* gene deletion mutant of RA-YM strain		This study
RAYM Δ*fur* (pRES-JXrep-spc-fur)	Complemented RA-YM Δ*fur* strain		This study
**PLASMIDS**			
pRE112	*SacB*,Cm^R^		This study
pRE-lacZ-mpheS-spc	Cm^R^,Spc		This study
pRES-JXrep-spc	Cm^R^,Spc		This study
**PRIMERS FOR CONSTRUCTION OF VECTOR pRE-lacZ-mpheS-spc**			
S1L	5′-AGGATCCTGTCGACCATATGTCCTAACCTTTTGGTAATG-3′		This study
S1R	5′- AGGAAATTACAGATCTGAGGGGACAGGCGAGAGACGAT-3′		This study
S2L	5′- CTCAGATCTGTAATTTCCTGCATTTGCCTGT-3′		This study
S2R	5′-AGGATCCACTCGAGTCTATCTGTTTCTTTTCATTCTCTG-3′		This study
PrpsLF	5′- GGGGTACCACTTTATCCATTTATAAAACTACATCA-3′	rpsL Promoter	This study
PrpsLR	5′-ATCAATATACTCTAACATTTAATTGCTTTTATTTATTTTTAGTTTC-3′		This study
phesF	5′-GAAACTAAAAATAAATAAAAGCAATTAAATGTTAGAGTATATTGAT-3′	*mpheS* gene	This study
phesR	5′-TCTATAGTCAAAAGGATACCCATTAAAAATAAAAAAGGAAACT-3′		This study
spcL	5′-ATTTTTAATGGGTATCCTTTTGACTATAGAGGATCGATCT-3′	*spc* gene	This study
spcR	5′-GCTCTAGACAGTAGTTTTAAAAGTAAGCACCTG-3′		This study
rpsL-lacZ	5′-TCTCGAGAACTTTATCCATTTATAAAACTACATCA-3′	rpsL Promoter	This study
rpsR-LacZ	5′-ATCCGTAATCATGGTCATTTAATTGCTTTTATTTATTTTTAGTTTC-3′		This study
lacZL	5′-CTAAAAATAAATAAAAGCAATTAAATGACCATGATTACGGATTCA-3′	*lacZ* gene	This study
lacZR	5′-CGGGATCCATCCAAAAGTTTGTGTTTTTTAAATAGT-3′		This study
**PRIMERS FOR CONSTRUCTION OF THE COMPLEMENTED SHUTTLE PLASMID pRES-JXrep-spc**			
rep1	5′-CCCTCGAGAATGCTTTGTGTTCCTCCCTTGTCA-3′	Replicon and replicase gene	This study
rep2	5′-GTTTTCGTTCCACTGAACTTTAGGATTGTCTGCTTGCGCT-3′		This study
spcL1	5′-GACAATCCTAAAGTTCAGTGGAACGAAAACTCACGTT-3′	*spc* gene	This study
spcR1	5′-CGGGATCCCAGTAGTTTTAAAAGTAAGCACCTG-3′		This study
**PRIMERS FOR CONSTRUCTION OF THE MUTANT** Δ***fur*** **AND THE COMPLEMENTED STRAIN**			
Fur-L1	5′-CATGCATGCTTGGATTACGGTAGTTCTTGCTG-3′	Upstream of *fur*	This study
Fur-L2	5′-GTATAATTAGCCTCATAGGTACTATTATTTTCTAGATTTA-3′		This study
Fur-R1	5′-AAAATAATAGTACCTATGAGGCTAATTATACTCGTACTAAT-3′	Downstream of *fur* gene	This study
Fur-R2	5′-GGGGTACCATGGTTTCTCCCGTGAGGACTTT-3′		This study
Promoter-fur1	5′- GGGGTACC ATAAAGTAATATTGCTATATTTA-3′	promoter of*fur* gene	This study
Promoter-fur2	5′- GAGAACTACAAGGTA ATATTAAAAACTTAATTTTTA-3′		This study
Fur-inL	5′- TTAAGTTTTTAATATTACCTTGTAGTTCTCTTTCTATA-3′	Coding sequence of *fur* gene	This study
Fur-inR	5′- CATGCATGCAATAGCAAAAAATACTGGCAT-3′		This study
**PRIMERS FOR RT-PCR**			
03924L	5′-GAAATACACGCTGATAGATGGTT-3′	*RAYM_03924*	This study
03924R	5′-TACCGTGGGCGTTATCATCTTCA-3′		This study
09824L	5′-TCCAAGTAGGCAACCAACGAGTC-3′	*RAYM_09824*	This study
09824R	5′-TGATGACAAGGCAGGACCGAGGG-3′		This study
09774L	5′-ATGTCCACCTCCAACTTATCTTC-3′	*RAYM_09774*	This study
09774R	5′-GGTTATCATCTTTCCGTCCACTT-3′		This study
00365L	5′-TTTTGACCATATTAGCGAACCTAC-3′	*RAYM_00365*	This study
00365R	5′-TTGATGCTACAATCCGTATGCTC-3′		This study
04506L	5′-TATCATCGTTCCCAAGGAGGTTT-3′	*RAYM_04506*	This study
04506R	5′-TCAAACGAAGGGAGCGAGGTCAT-3′		This study
00965L	5′-CGTCTGTAGTGATGAGGGTTTGA-3′	*RAYM_00965*	This study
00965R	5′-CTATGTATTTGGCTTTATCCCTTC-3′		This study
01847L	5′-CGTTACTTATCATCGGAACTGGA-3′	*RAYM_01847*	This study
01847R	5′-AGCCAGCATTTCGTTAGAGTTAT-3′		This study
06180L	5′-GAGTGCCTACCACCGAATA-3′	*RAYM_06180*	This study
06180R	5′-TGGCAGGTGTAAGGTACGATTA-3′		This study
**PRIMERS FOR EMSA**			
Biotin-06180F	5′-CTATTTTGTTAGGCTGTTCCTCCAC-3′	Promoter of *RAYM_06180*	This study
Biotin-06180R	5′-GAACTTTGCCCCAATAGAGGTAATC-3′		This study
Biotin-01847F	5′-AAAGATGGTAAAGTAGCTAGCCCTG-3′	Promoter of *RAYM_01847*	This study
Biotin-01847R	5′-CGCCGAAGCTAATAGTATAAGAGGT-3′		This study
Biotin-03924L	5′-AGATTACTATAACGCCGTTCTTC-3′	Promoter of *RAYM_03924*	This study
Biotin-03924R	5′-ATAATAAGTGTTAGGCGTTGGGT-3′		This study
Biotin-09824L	5′-CCCTGCGACACGACCTTCTAACA-3′	Promoter of *RAYM_09824*	This study
Biotin-09824R	5′-ACCACAACGGAACAACTACAGGA-3′		This study

### Construction of suicide vector pRE-lacZ-mpheS-spc and complemented shuttle vector pRES-JXrep-spc

For the construction of suicide vector pRE-lacZ-mpheS-spc, a 3.9 kb fragment of pRE was amplified from suicide vector pRE112 using primer S1L (Table 1) (introducing *BamH*I, *Sal*I, and *Nde*I site) and S2R (Table 1) (introducing *Xho*I and *BamH*I site); then digested with *BamH*I enzyme and ligated to generate circular pRE which contained the essential components of the conjugational transfer. As RA cannot catabolize sucrose, the selected marker *SacB* was removed. The mutated *pheS* gene (*mpheS*) and the sequence of the multiple cloning sites were engineered into pUC57 to generate pUC57-mpheS and pUC57-MCS by the GenScript Corporation (Nanjing, China). This pUC57-mpheS vector contained the mutated *R. anatipestifer pheS* gene with altered DNA sequences (Supplemental Figure 1), which was driven by an upstream PS12 promoter of the *R. anatipestifer rpsL* gene. The 1.1 kb *spc* cassette was amplified from plasmid pIC333 using primers spcL and spcR (Table 1), *mpheS* was amplified from plasmid pUC57-mpheS using primers pheS1and pheS2 (Table 1). The *spc* cassette was then fused with the *mpheS* fragment using overlap PCR (introducing *Kpn*I and *Xba*I site). The fragment of mpheS-spc was inserted into pMD18T to generate pMD18T-mpheS-spc. The pMD18T-mpheS-spc was digested with *Kpn*I and *Xba*I enzymes, and 2.2 kb PS12-mpheS-spc fragment was ligated into pUC57-MCS and digested with *Kpn*I and *Xba*I to obtain pUC57-MCS-mpheS-spc. The pRE and pUC57-MCS-mpheS-spc were digested with *Xho*I and *Xba*I and ligated to generate pRE-mpheS-spc. The next step was to amplify a 3.3 kb fragment of *lacZ* from *E. coli* BL21 genome using primer lacZR and lacZL (Table 1) (introducing a *BamH*I site). A 135 bp fragment of PS12 promoter was amplified from RA-YM using primer rpsL-LacZ (introducing a *Xho*I site) and rpsR-LacZ (Table 1) and fused to *lacZ* fragment by overlap PCR using rpsL-LacZ and lacZR primers. pRE-mpheS-spc and *lacZ* fragments were digested with *BamH*I and *Xho*I and ligated to obtain 9 kb pRE-lacZ-mpheS-spc.

The shuttle plasmid pRES-JXrep-spc was constructed in several steps including the amplification of 2.5 kb fragment of replicon region and replicase gene (Genebank: KY806579) with primers rep1 and rep2 (Table 1) (introducing *a Xho*I site). The wild type plasmid of *R. anatipestifer* strain RA-JX was used as a template. A 1.1 kb *spc* cassette was amplified from plasmid pIC333 using primers spcL1 and spcR1 (Table 1), thereby introducing a *BamH*I site. The replicon region of RA-JX was joined with the *spc* cassette using overlap PCR. The JXrep-spc fragment was inserted into pMD18T to obtain pMD18T-JXrep-spc. In the next step, plasmid pRE and pMD18T-JXrep-spc were digested with *Xho*I and *BamH*I and ligated to generate the shuttle plasmid pRES-JXrep-spc.

### Construction of unmarked deletion *R. anatipestifer* Δ*fur* and complemented mutant strains

To obtain the suicide vector pRE-lacZ-mpheS-spc-fur for the deletion of whole *fur* gene from *R. anatipestifer* RA-YM strain, upstream (738 bp) and downstream (802 bp) DNA fragments were amplified using primers Fur-L1 and Fur-L2 (Table 1) (introducing *Sph*I site), Fur-R1 and Fur-R2 (Table 1) (introducing *Kpn*I site), respectively. The two fragments were joined together by overlap PCR. The LR fragment and pRE-lacZ-mpheS-spc were digested with *Sph*I and *Kpn*I; 1.5 kb LR fragment was inserted into pRE-lacZ-mpheS-spc to generate the suicide vector pRE-lacZ-mpheS-spc-fur. *E. coli* strain x7213 was used as a donor in conjugation step to introduce the suicide vector pRE-lacZ-mpheS-spc-fur into RA-YM strain as described previously (Hu et al., 2011). For phenotypic detection of mutant strains, conjugation filters were plated on tryptic soya agar (TSA) containing 100 μg/mL Spc. Colonies were then grown on TSA containing cPhe (0.2%) and X-gal (40 μg/mL). Appearance of white colonies confirmed successful construction of deletion mutant strains. For identification of recombinants carrying the chromosomal *fur* gene deletion, colonies were analyzed using PCR primers FurL1 and FurR2 to determine presence of wild-type or mutant allele at the target locus. The wild-type and deleted alleles could be differentiated on the basis of size of amplicon by agarose gel electrophoresis.

Similarly, for generation of complemented mutant strain, shuttle vector pRES-JXrep-spc-fur was constructed by amplification of the promoter sequence (171 bp) and the coding sequence (486 bp) of *fur* gene. The promoter sequence and the coding sequence were amplified using primers Promoter-fur1 and Promoter-fur2 (introducing *Kpn*I site, Table 1), primers Fur-inL and Fur-inR (introducing *Sph*I site, Table 1). The two fragments were joined together by overlap PCR. The plasmid pRES-JXrep-spc and *fur* gene fragment were digested with *Sph*I and *Kpn*I, then the fragment of 657 bp was inserted into plasmid pRES-JXrep-spc to obtain shuttle vector pRES-JXrep-spc-fur. The *E. coli* strain x7213 was used as donor in conjugation transfer of shuttle vector into the RA-YM Δ*fur* strain (Hu et al., 2011). The phenotypic identification of complemented mutant strain was conducted on TSA plates containing Spc 100 μg/mL. Furthermore, PCR reaction was performed using primers Fur inL and Fur inR to ensure that recombinant strains were harboring shuttle vectors.

### RNA-sequencing of wild-type and RA-YM Δ*fur* deletion mutant in iron-restricted and enriched conditions

The colonies of the wild-type and the RA-YM Δ*fur* mutant were suspended into tryptic soya broth (TSB) and incubated overnight with shaking at 37°C to an OD_600_ of 0.2. FeCl_3_ (Sigma-Aldrich) or 2, 2-Dipyridyl (2, 2-DP, Sigma-Aldrich) was added to the bacterial suspension to produce a final concentration of 200 and 30 μM, as iron restricted and iron rich conditions, respectively and incubated at 37°C until the OD_600_ reached 0.8. Total RNA was extracted from bacteria solution using Bacterial RNA Kit (OMEGA, Norcross, USA) following the guidelines. Extracted RNA was purified with RNase-free DNase (Promega, Wisconsin, USA) at 37°C for 30 min to remove impurities of DNA, the DNA-free purified RNA was examined by 1% agarose gel electrophoresis. Purified RNA (23S rRNA and 16S rRNA) was sent to Huada Gene Center (Shenzhen, China) for RNA sequencing. All RNA samples were performed in two independent biological replicates (BioProject: SRP106941).

### Quantitative reverse transcription PCR (RT-qPCR)

RT-qPCR was performed to quantify the expression of genes regulated by Fur. Primers were designed with Primer 5.0 software. RNA was extracted from wild-type and RA-YM Δ*fur* strains grown in iron-restricted and iron-rich medium. RNA was reverse transcribed to cDNA using PrimerScript RT regent Kit with gDNA Eraser (Takara, Dalian, China). Real-time PCR reaction was performed using SYBR Premix (Takara, Dalian, China). Each reaction was performed in triplicate. Relative quantification of gene expression was calculated according to 2^−ΔΔCt^ method, RA-YM 16S rRNA was used as reference gene for normalized expression for each RNA sample.

### The expression of fur protein and electrophoretic mobility-shift assay (EMSA) of the putative fur-box sequence

The fragment of *fur* gene was amplified using the primers Fur1 and Fur2 (introducing the *BamH*I and *Xho*I sites); the fragment and vector pET-28a were digested with *BamH*I and *Xho*I and restricted fragment was ligated into the expression vector pET-28a to generate the expression vector pET-28a-fur. The expression plasmid was then transformed into competent cells of *E. coli* BL21 (DE3). Then the Fur protein was purified with an ÄKTA Purifier (GE His Trap FF, USA).

EMSA was performed with the Lightshift Chemiluminescent EMSA Kit (Thermo fisher scientific, Waltham, USA). The reaction was incubated at 30°C for 1 h, then loaded into 6% non-denaturing polyacrylamide gel electrophoretic and exposured. The reaction mixture (20 μL) contained 1 μg biotin labeled DNA fragment, 2 μL binding buffer, 1 μL KCl, 1 μL MgCl_2_, 1 μL glycerol, 1 μL NP-40 and 1 μL Poly(dI-dC) and desired concentration of Fur protein, the final concentration of Fur protein were 0, 0.1, 1, and 10 μg in four lanes. 16S rDNA was used as a negative control. DNA fragments to be identified were amplified by biotin labeled primer (Sangon, Shanghai, China). The length of DNA fragments ranged from 350 to 420 bp.

### Assessment of virulence *in vivo*

One-day-old Cherry Valley ducklings obtained from the Wuhan Duck Farm (Wuhan, China) housed in cages under 12-h light/dark cycle, at controlled temperature (28–30°C) and free access to food and water during the whole course of this study. Care and maintenance of all animals were in line with the standards of Institutional Animal Care. This experiment was approved by the Institutional Animal Experimental Committee of the Veterinary Faculty of Huazhong Agricultural University.

To determine the role of *fur* in virulence, the median lethal dose (LD_50_) of the deletion mutant RA-YM Δ*fur* strain, the complemented mutant RA-YM Δ*fur* strain and the wild-type RA-YM was measured using the Reed–Muench method (Reed and Muench, 1938). For each wild type, mutant and complemented strains, 12-day-old ducklings were evenly divided into five groups (10 ducklings/group). All five groups were injected intramuscularly with 1.0 × 10^4^, 1.0 × 10^5^, 1.0 × 10^6^, 1.0 × 10^7^, and 1.0 × 10^8^ colony forming units (CFU) of wild type strain, respectively. Similarly, mutant and complemented strains were injected to respective groups of ducklings for the evaluation of LD_50_. Moribund ducklings were killed humanely and counted as dead. Dead ducklings were identified for the presence of RA. Mortality of the ducklings was recorded daily for a period of 10 days.

A comparative analysis of bacterial load in the blood of ducklings infected with mutants and wild type was made. Blood and target organs (brain, liver, heart and spleen) were collected at 24 and 48 h post-inoculation (five ducklings per group at each time-point). The target organs were homogenized with PBS to obtain supernatant. Blood and supernatant were plated on TSB agar plates for bacterial count with a 10-fold dilution method. In addition, the degree of lesions developing on the liver, spleen, heart and brain by the wild-type and the Δ*fur* mutant strains were also recorded. For pathological investigations, all tissues were immersed in 10% formalin solution, embedded in paraffin section and stained with hematoxylin and eosin (H E). The pathological findings of the wild-type and the RA-YM Δ*fur* mutant were compared.

## Results

### Characterization of *R. anatipestifer* Δ*fur* mutant and RA-YM Δ*fur* complemented mutant strain

**Figure 1:**
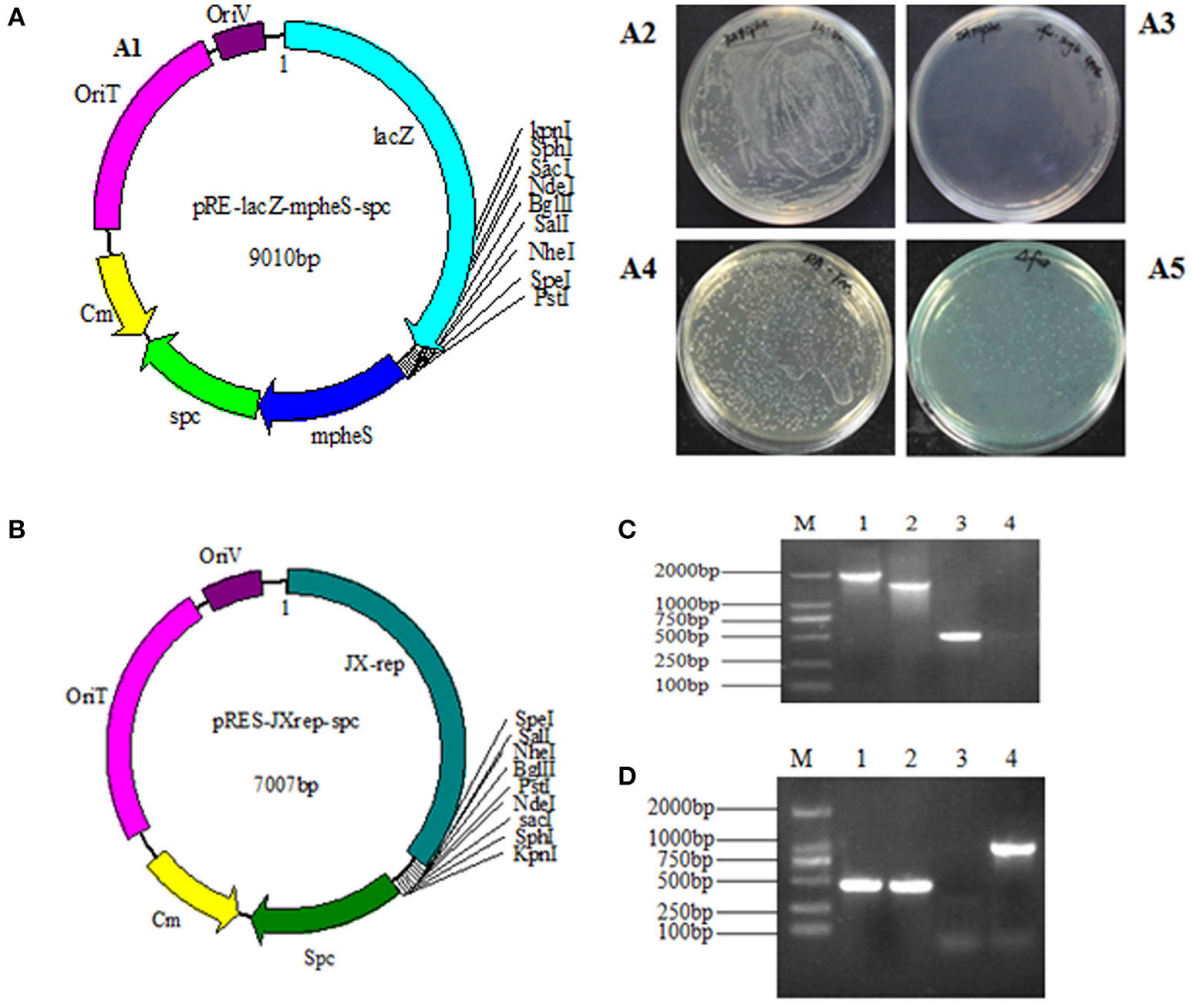
**(A1)** The map of plasmid pRE-lacZ-mpheS-spc containing resistance gene *spc*, counter-selectable marker *mpheS* and *lacZ*; **(A2,A3)** show the growth of the wild type strain and the merodiploid strains on the TSA plate with 0.2% cPhe respectively. **(A4)** The color of the RA-YM on X-gal plate was white. **(A5)** The color of the merodiploid strains on X-gal plate was blue. **(B)** The map of the complemented shuttle plasmid pRES-JXrep-spc. **(C)** The PCR amplification of the RA-YM Δ*fur* deletion mutant strain and wild type RA-YM strain. Lane M: DL2000 DNA Marker; Lane 1: LR fragment amplification from RA-YM; Lane 2: LR fragment amplification from RA-YM Δ*fur* deletion mutant strain; Lan 3: Amplification of *fur* gene from RA-YM; Lane 4: Amplification of *fur* gene from RA-YM Δ*fur* deletion mutant strain; **(D)** The PCR amplification of RA-YM Δ*fur* mutant complemented strain. Lane M: DL2000 DNA Marker; Lan 1: Amplification of *fur* gene wild type RA-YM; Lane 2: Amplification of *fur* gene RA-YM Δ*fur* complemented mutant strain; Lane 3: Amplification of *spc* gene from wild type RA-YM; Lane 4: *spc* gene amplification from the RA-YM Δ*fur* complementary mutant strain.

As homologous recombination follows a two-step procedure, the selection of the *R. anatipestifer* Δ*fur* mutant was carried out in two steps (Stibitz, 1994). The selection of mutant with Spc resistance was initially carried out, followed by the expression of *mpheS* gene. The function of *lacZ* gene could directly confirm whether the plasmid had been excised. The *pheS* gene was engineered by substituting alternative bases at numerous positions. The sequence similarity rate between wild-type *pheS* and *mpheS* was 71%. However, no difference was observed in amino acid sequence with the exception of the A301G mutation. In this study, the *mpheS* gene was driven by the promoter of RA *rpsL* gene. The first process was obtained by growing RA-YM strains on TSA medium containing Spc resistance. The first process obtained the merodiploid strains, which harbored the suicide vector. Then, the merodiploid strains were screened on TSA medium containing 0.2% cPhe and X-gal to obtain the deletion mutant. The merodiploid strains grew on the plate containing Spc but had no growth on the agar plate with 0.2% cPhe. The wild strains could be grown on the plate contains cPhe (Figures 1A2,A3). In addition, the merodiploid strains appeared as blue colonies while the wild type strain was of a white color on the plate containing X-gal (Figures 1A4,A5). This finding demonstrated the effectiveness of the counter-selectable markers *pheS* in RA. A suicide vector pRE-lacZ-mpheS-spc-fur, containing mutated *pheS* as a counter-selectable marker (Figure 1A1) was constructed and successfully transformed into RA-YM strain to generate RA-YM Δ*fur* strain.

To determine whether the plasmid replicated and deleted *fur* gene after homologous recombination, no PCR amplification of *fur* gene and smaller LR fragment size (1,540 bp) from RA-YM Δ*fur* strain as compared to larger LR fragment size (2,008 bp) from RA-YM strain (Figure 1C) confirmed the plasmid activity and recombination. Similarly, development of a recombinant RA-YM Δ*fur* complemented mutant strain was confirmed by PCR amplification of *fur* and *spc* genes as shown in Figure 1D.

### Transcriptional response in iron enriched and restricted environment

A comparison of gene expression regulated by iron and/or Fur in wild type (WT) and mutant (Δ*fur*) strains grown in iron rich (+Fe) and iron restricted condition(−Fe) was exclusively established and comparison of RA-YM Δ*fur* deletion mutant strain with wild type RA-YM was also performed. In our experiments, the significance of differentially expressed genes was estimated by the false discovery rate (FDR) and was considered significant if FDR < 0.001 and the |log_2_Ratio| >1(Ernst et al., 2005a; Ledala et al., 2010). In total, 25 genes were downregulated and 45 genes were upregulated by iron when grown in iron-restricted conditions, in both parent and mutant. Seventeen genes were directly regulated by Fur. The expression of eight genes randomly selected regulated by iron and Fur was confirmed by real-time PCR in RA-YM and the Δ*fur* mutant (Figure 7). The real-time PCR result was in accordance to the transcriptional data.

#### Downregulation of genes by iron

Exclusively, 25 genes were downregulated when grown without iron in medium in both wild type RA-YM and RA-YM Δ*fur* deletion mutant strains. Furthermore, ratio (WT-Fe/WT+Fe ratio and Δ*fur*-Fe/Δ*fur*+Fe ratio) of gene expression were calculated in both iron-restricted (−Fe) and iron-rich (+Fe) conditions, which were ≥2. Of the 25 downregulated genes, five genes encoded proteins which acted as transporters; six genes encoded enzymes which participated in tricarboxylic acid cycle; six were involved in oxidation-reduction; six genes encoded hypothetical proteins and two genes actively participated in amino acid biosynthesis (Table 2).

**Table 2:** Genes downregulated by iron in response to iron restricted condition.

**Gene name**	**Predicted function**	**Ratio**			
		**WT-Fe/WT+Fe**	**Δ*fur*-Fe/Δ*fur*+Fe**	**Δ*fur*-Fe/WT-Fe**	**Δ*fur*+Fe/WT+Fe**
**ENERGY METABOLISM**					
*RAYM_00925*	Fumarate hydratase	−1.26	−2.78	−1.54	
*RAYM_01977*	Succinate dehydrogenase cytochrome b subunit, b558 family	−1.01	−2.47		
*RAYM_08220*	Aconitase	−1.56	−1.42		
*RAYM_01982*	Succinate dehydrogenase flavoprotein subunit	−1.04	−2.75		
*RAYM_01987*	Succinate dehydrogenase iron-sulfur subunit	−1.46	−2.79		
*RAYM_02307*	NADH-ubiquinone oxidoreductase chain G	−1.66	−1.72	−1.09	
**TRANSPORTER**					
*RAYM_02992*	Efflux transporter, RND family, MFP subunit	−2.33	−2.70		
*RAYM_04896*	TonB-dependent outer membrane receptor	−1.94	−2.57		−2.17
*RAYM_04891*	Amino acid/peptide transporter	−1.25	−1.64		
*RAYM_00465*	Co/Zn/Cd efflux system membrane fusion protein	−1.38	−1.56		
*RAYM_02982*	Integral membrane protein	−1.39	−1.82		
**OXIDATION-REDUCTION**					
*RAYM_00020*	Cytochrome c oxidoreductase quinone-binding subunit 1	−1.01	−3.47	−2.23	
*RAYM_03017*	Cytochrome c551/c552	−1.32	−1.57		
*RAYM_01530*	Cytochrome c oxidase subunit CcoP	−1.12	−1.07		
*RAYM_01540*	Cytochrome c oxidase subunit CcoN	−1.10	−1.09		
*RAYM_07584*	Cytochrome c nitrate reductase, small subunit	−2.06	−1.36		
*RAYM_07589*	Nitrite reductase (cytochrome; ammonia-forming)	−1.01	−1.01		
**BIOSYNTHESIS OF CYSTEINE**					
*RAYM_04786*	Cysteine synthase A	−3.23	−1.27		−2.50
*RAYM_04791*	Serine O-acetyltransferase	−2.10	−1.40	−1.45	−1.95
**HYPOTHETICAL PROTEIN**					
*RAYM_05775*	Hypothetical protein	−1.24	−1.09	1.37	
*RAYM_05780*	Hypothetical protein	−2.36	−1.67	1.49	
*RAYM_00895*	Hypothetical protein	−2.03	−1.01		
*RAYM_03012*	Hypothetical protein	−1.69	−1.34		
*RAYM_03172*	Hypothetical protein	−2.26	−1.13	2.15	
*RAYM_01690*	Hypothetical protein	−1.63	−2.39		

#### Upregulation of genes by iron

Similarly, 45 genes were upregulated by iron both in the parent and the Δ*fur* mutant strain when iron was restricted. Gene ratio (WT-Fe/WT+Fe ratio and Δ*fur*-Fe/Δ*fur*+Fe ratio) was calculated ≥2. Among the 45 upregulated genes, nine genes participated in amino acids and cofactor biosynthesis; six were involved in cell envelope and surface structure formation; eight genes were involved in protein synthesis; transport and binding protein were encoded by six genes; three genes encoded regulators; cellular processes were regulated by three genes, and ten encoded hypothetical proteins (Table 3).

**Table 3:** Genes upregulated by iron in response to iron restricted condition.

**Gene name**	**Predicted function (gene)**	**Ratio**			
		**WT-Fe/WT+Fe**	**Δ*fur*-Fe/Δ*fur*+Fe**	**Δ*fur*-Fe/WT+Fe**	**Δ*fur*+Fe/WT+Fe**
**BIOSYNTHESIS OF AMINO ACIDS, COFACTORS, AND PROSTHETIC GROUPS**					
*RAYM_04219*	Phosphoserine aminotransferase	1.35	1.25		
*RAYM_04224*	D-3-phosphoglycerate dehydrogenase	1.35	1.19		
*RAYM_04506*	Thiamine biosynthesis protein ApbE	1.84	3.37		
*RAYM_06215*	Aminodeoxychorismate lyase	1.08	1.12		
*RAYM_06377*	Para-aminobenzoate synthase component I	1.53	1.01		
**CELL ENVELOPE AND SURFACE STRUCTURES**					
*RAYM_01390*	GtrA family protein	2.00	1.80	−1.26	
*RAYM_02732*	Lipid A biosynthesis lauroyl acyltransferase	1.15	1.18		
*RAYM_04004*	Monofunctional biosynthetic peptidoglycan transglycosylase	1.70	1.31		−1.31
*RAYM_08765*	ATPase YjeE, predicted to have essential role in cell wall biosynthesis	1.13	1.21		
*RAYM_06482*	Outer membrane lipoprotein nlpE	1.28	2.45	1.14	
**CELLULAR PROCESSES**					
*RAYM_00990*	Non-specific DNA-binding protein Dps	2.00	3.24		
*RAYM_01160*	Ferritin	2.02	2.74		
*RAYM_04501*	Nitric oxide synthase	2.80	3.73		
**DNA METABOLISM, RESTRICTION AND MODIFICATION**					
*RAYM_00455*	Predicted DNA alkylation repair enzyme	1.99	1.14		
**FATTY ACID AND PHOSPHOLIPID METABOLISM AND BIOSYNTHESIS**					
*RAYM_04164*	Isopentenyl diphosphate isomerase	1.38	1.26		
**HYPOTHETICAL PROTEINS/UNKNOWN FUNCTION**					
*RAYM_00065*	Hypothetical protein	1.19	1.09		
*RAYM_00865*	Hypothetical protein	1.57	1.61		
*RAYM_04229*	Hypothetical protein	1.35	1.10		
*RAYM_04491*	Hypothetical protein	5.24	5.32		
*RAYM_04496*	Hypothetical protein	3.42	4.35		
*RAYM_05980*	Hypothetical protein	1.41	1.65		
*RAYM_06462*	Four helix bundle protein	1.41	2.09		
*RAYM_06477*	Hypothetical protein	1.43	2.49	1.32	
*RAYM_06862*	Rare lipoprotein A	3.23	1.83		
*RAYM_09774*	Leucine-rich repeat-containing protein	3.05	2.83		
**PROTEIN SYNTHESIS**					
*RAYM_00750*	RNA polymerase Rpb6	2.24	1.43	−1.08	
*RAYM_01100*	Nitrogen-fixing NifU domain protein	1.87	1.15		
*RAYM_01495*	FeS assembly SUF system protein	1.27	1.71		
*RAYM_06457*	Probable iron binding protein from the HesB_IscA_SufA family	1.53	2.58	1.04	
*RAYM_06467*	Cysteine desulfurase activator complex subunit SufB	1.87	1.85		
*RAYM_06507*	FeS assembly protein SufD	1.56	1.78		
*RAYM_03082*	Protein-(glutamine-N5) methyltransferase, release factor-specific	1.27	1.99		
*RAYM_03087*	tRNA methyltransferase	1.42	1.63		
**PURINES, PYRIMIDINES, NUCLEOSIDES, AND NUCLEOTIDES**					
*RAYM_03724*	Orotate phosphoribosyltransferase	2.10	3.30		
*RAYM_06492*	5-hydroxyisourate hydrolase	1.22	1.84	1.00	
**REGULATORY FUNCTIONS**					
*RAYM_00365*	RNA polymerase sigma-70 factor, ECF subfamily protein	1.93	1.51	−1.05	
*RAYM_07184*	Transcriptional regulator	1.61	1.25		
*RAYM_08270*	Transcriptional regulator, XRE family	1.02	1.05		
**TRANSPORT AND BINDING PROTEINS**					
*RAYM_00510*	Ferrous iron transport protein A	1.52	1.03	−2.75	−2.25
*RAYM_00515*	Ferrous iron transport protein B	1.33		−3.03	−2.22
*RAYM_04481*	TonB-dependent receptor	6.36	5.49		
*RAYM_06602*	Outer membrane efflux protein	1.07	1.05		
*RAYM_06607*	ABC transporter related protein	1.86	1.51		
*RAYM_06472*	Accessory colonization factor AcfC	1.14	2.15		

#### Gene regulation by fur under iron restricted conditions

In almost all bacteria, Fur acted as a negative regulator. Genes directly regulated by Fur were observed to be upregulated when iron was restricted and when *fur* was mutated. The ratio (WT-Fe/WT+Fe ratio, Δ*fur-*Fe/Δ*fur*+Fe ratio and Δ*fur*+Fe/WT+Fe ratio) of 17 genes regulated by Fur was ≥2 (Table 4). Of the 17 genes directly regulated by Fur, five genes contributed in iron acquisition; two were involved in oxidation-reduction; one gene participated in activation of type IX secretion system (T9SS); the functions of six genes remained unknown, and three encoded hypothetical proteins (Table 4).

**Table 4:** Putative genes regulated by Fur under iron restricted condition.

**Gene name**	**Predicted function**	**Ratio**			
		**WT-Fe/WT+Fe**	**Δ*fur*-Fe/Δ*fur*+Fe**	**Δ*fur*-Fe/WT-Fe**	**Δ*fur*+Fe/WT+Fe**
*RAYM_00450*	Oxidoreductase	3.31	3.67	1.67	1.73
*RAYM_01847*	TonB-dependent outer membrane protein receptor for Fe^3+^-dicitrate	2.22	1.94	1.96	1.63
*RAYM_03589*	Rhodanese-like domain protein	1.63	2.18	1.77	1.21
*RAYM_03864*	3-hydroxyacyl-CoA dehydrogenase/Enoyl-CoA hydratase	1.44	2.01	1.76	1.20
*RAYM_03869*	Regulatory protein, MarR	2.48	4.05	1.71	1.37
*RAYM_03924*	SprT protein	1.01	1.17		1.08
*RAYM_05635*	L-asparaginase	1.76	1.26		1.30
*RAYM_06175*	Hypothetical protein (HmuY)	6.71	7.69	2.53	2.56
*RAYM_06180*	Outer membrane receptor for ferrienterochelin and colicins	7.89	8.44	1.93	2.64
*RAYM_06185*	Hypothetical protein	7.25	8.37	2.18	2.25
*RAYM_07324*	Mammalian cell entry protein	1.46	1.32	1.01	1.06
*RAYM_07989*	Hypothetical protein	1.14	1.59		2.36
*RAYM_09779*	TonB-dependent receptor	2.11	2.70		1.90
*RAYM_09784*	Vitamin K-dependent gamma-carboxylase	2.64	2.77		1.39
*RAYM_09789*	Putative lipoprotein Imelysin	3.07	2.92		1.93
*RAYM_09794*	Hypothetical protein	2.41	3.07	1.14	1.36
*RAYM_09824*	Putative outer membrane protein, mostly Fe transport	2.41	3.06	2.14	1.36

### Identification of putative binding sequences of fur-box binding to fur protein

The putative Fur binding sequence and the distance from the start condon of the genes regulated by Fur is shown in Table 5. Promoter sequences were analyzed using software RegPredict and ClustalW for identification of putative binding sequence of Fur protein which was 19 bp long and sequence was predicted as 5′-ATTTAGAATTATTCTAAAT-3′ (Figure 8A). Therefore, the Fur binding sequence might be located within 100 bp of the translation initiation codon of the regulated genes (Table 5). To verify the putative role of the Fur-box sequence, the promoters of *hmuR, sprT, RAYM_01847, RAYM_09824* were selected for electrophoretic mobility shift assay (EMSA). Our findings illustrated that purified Fur protein could bind to the DNA fragment containing the putative Fur-box (Figure 8B).

**Table 5:** Identification of putative Fur binding sequences (Fur boxes).

**Locus ID**	**Nucleotide position**		**Fur box sequence**	**Predicted function (Gene name)**	**ATG-distance**
	**Start**	**End**			
*RAYM_00450*	91412	91430	ATTTAGAATAATTAAAAAA	Oxidoreductase	9
*RAYM_01847*	9623	9641	ATTTAGAATTATCCTAAAT	Outer membrane receptor for Fe^3+^-dicitrate	67
*RAYM_03589*	76340	6358	ATTTAGAATTAGAATAAAT	Rhodanese-like domain protein	30
*RAYM_03869*	79344	79362	ATTTATAATATTGATTATT	Regulatory protein, MarR	87
*RAYM_03924*	86294	86312	AATGATAAACACTTTAACT	SprT protein	85
*RAYM_05635*	94340	94358	GTTTAAAATTTATCTAATT	L-asparaginase	27
*RAYM_06180*	205354	205372	ATTTAAAATTATTCTAAAT	HmuR	78
*RAYM_06185*	205354	205272	ATTTAGAATAATTTTAAAT	Hypothetical protein	25
*RAYM_07324*	79551	79569	ATTTATATTTATTTTTGAT	Mammalian cell entry protein	86
*RAYM_07989*	205419	205437	ATTTATTTTCAGTTTTAAT	Hypothetical protein	91
*RAYM_09824*	15328	15346	ATTTATACTTATTCTAATT	Putative outer membrane protein, mostly Fe transport	33

### *In vivo* evaluation of virulence of *R. anatipestifer* Δ*fur* deletion mutants

The LD_50_ values of RA-YM, RA-YM Δ*fur* deletion mutant and RA-YM Δ*fur* complemented strain were recorded as 2.0 × 10^6^ CFU, 1.6 × 10^8^ CFU, 1.2 × 10^7^CFU, respectively. LD_50_ counted for wild type as compared to RA-YM Δ*fur* deletion mutant was approximately 80 times higher, whereas no significant difference was observed as in the case of wild type in comparison to RA-YM Δ*fur* complemented mutant that was six times higher. Due to slight difference between wild type and RA-YM Δ*fur* complemented mutant, a further comparison was established only between wild type and RA-YM Δ*fur* deletion mutant strains. As the *fur* gene was disrupted in RA-YM Δ*fur* deletion mutant strains resulted in attenuation of virulence of RA. However, virulence to ducklings was partially restored when the mutant was complemented with the plasmid pRES-JXrep-spc.

**Figure 2:**
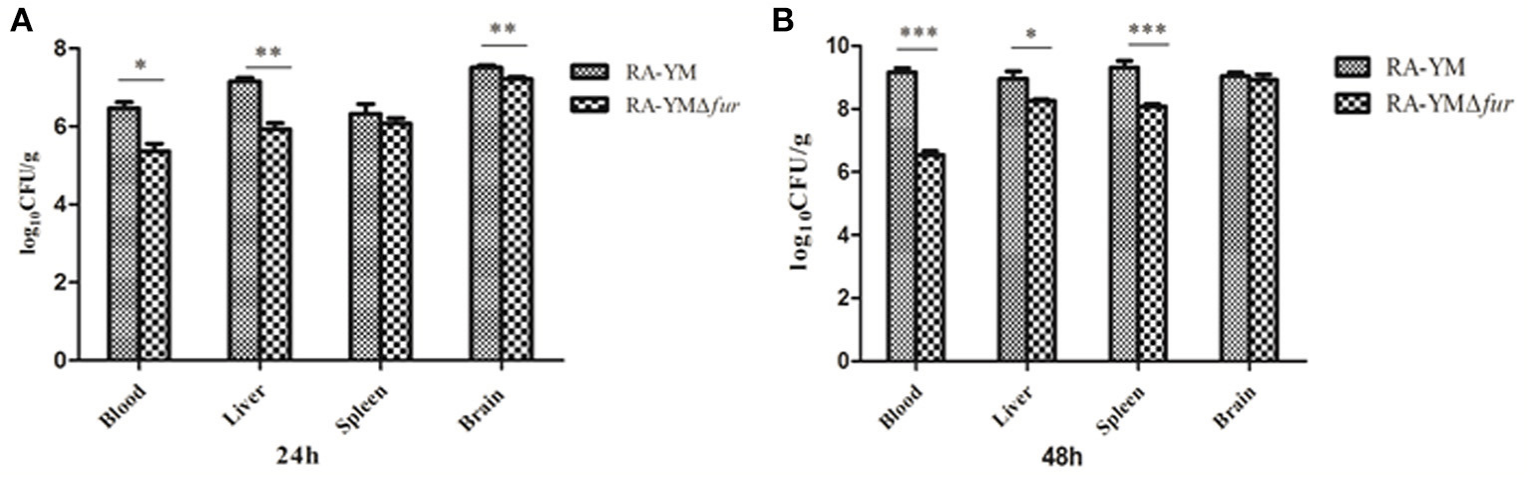
Graphical presentation of bacterial load in blood, liver, spleen, and brain of ducklings infected with wild type RA-YM and RA-YM Δ*fur* deletion mutant strain. The error bars represent mean ± standard deviation from five ducks. **(A)** The tissue burden of the group infected with wild type and RA-YM Δ*fur* deletion mutant after 24 h. **(B)** The tissue burden of the group infected with wild type and RA-YM Δ*fur* deletion mutant after 48 h.

**Figure 3:**
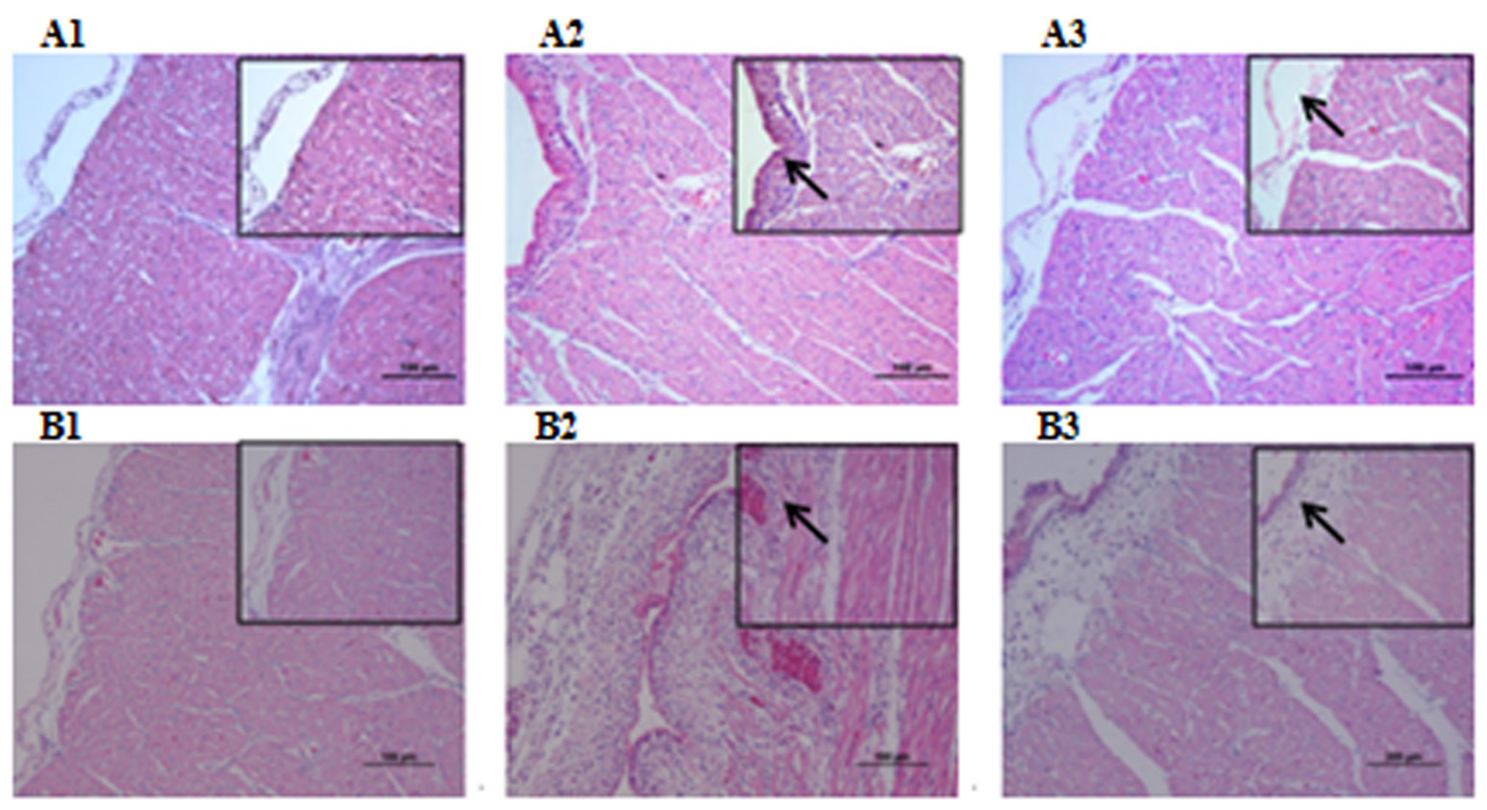
Histopathological diagram of heart. **(A1)** The blank control group after 24 h, **(A2)** The group with wild type RA-YM strain after 24 h, **(A3)** The group with RA-YM Δ*fur* deletion mutant strain after 24 h. **(B1)** The blank control group after 48 h, **(B2)** The group with wild type RA-YM strain after 48 h, **(B3)** The group with RA-YM Δ*fur* deletion mutant strain after 48 h.

**Figure 4:**
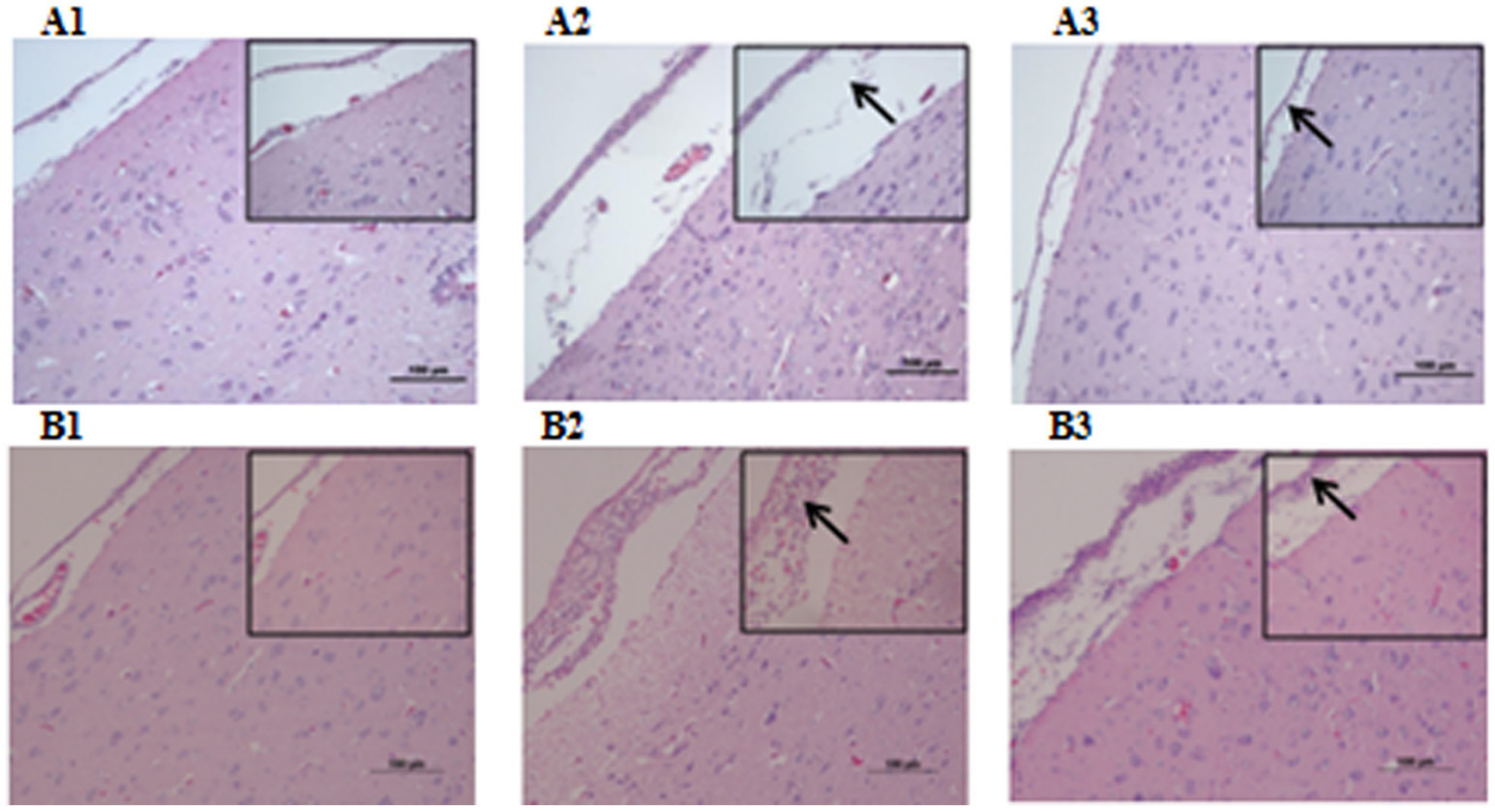
Histopathological diagram of brain **(A1)** The blank control group after 24 h, **(A2)** The group with wild type RA-YM strain after 24 h, **(A3)** The group with RA-YM Δ*fur* deletion mutant strain after 24 h. **(B1)** The blank control group after 48 h, **(B2)** The group with wild type RA-YM strain after 48 h, **(B3)** The group with RA-YM Δ*fur* deletion mutant strain after 48 h.

**Figure 5:**
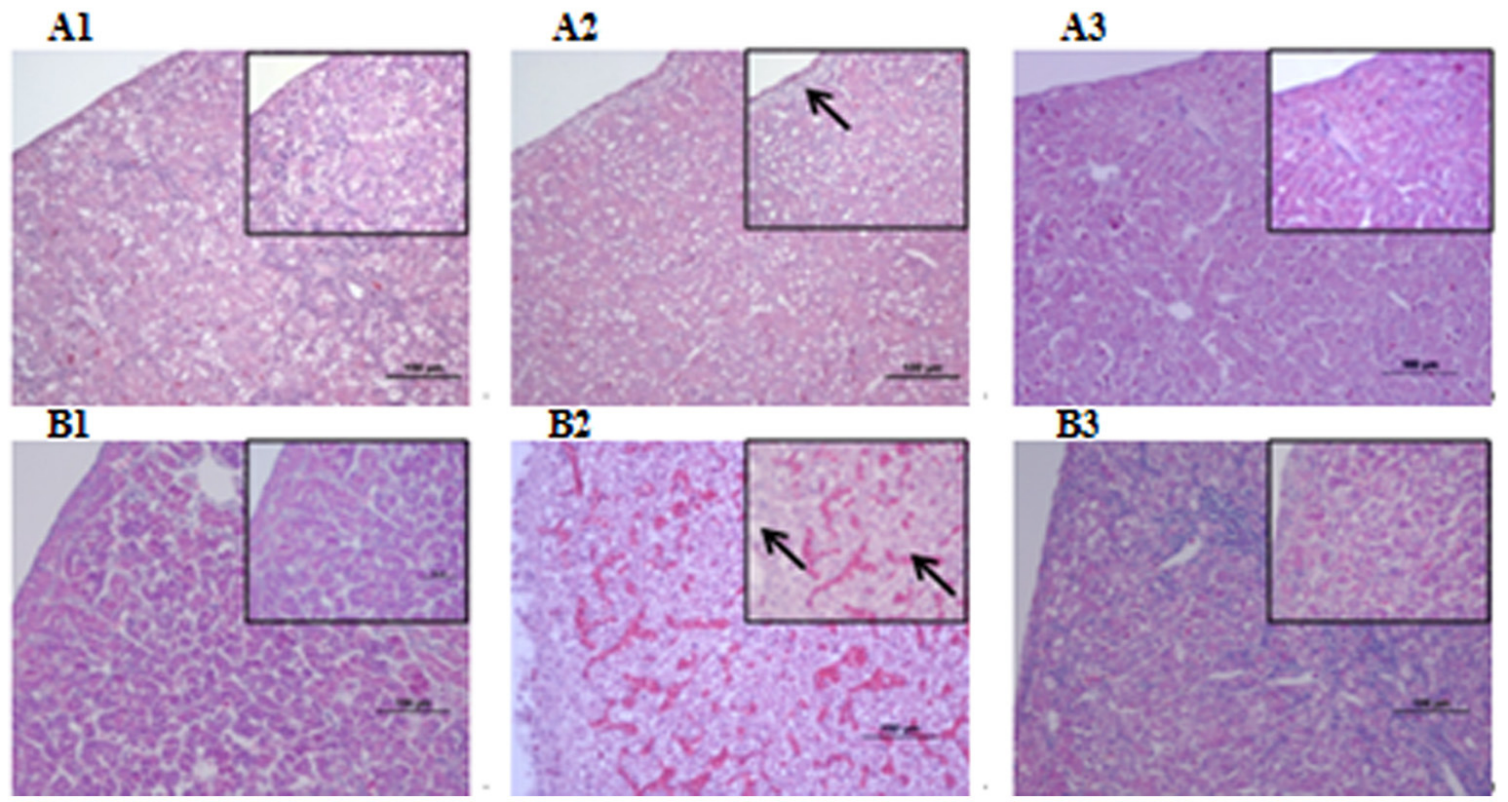
Histopathological diagram of liver **(A1)** The blank control group after 24 h, **(A2)** The group with wild type RA-YM strain after 24 h, **(A3)** The group with RA-YM Δ*fur* deletion mutant strain after 24 h. **(B1)** The blank control group after 48 h, **(B2)** The group with wild type RA-YM strain after 48 h, **(B3)** The group with RA-YM Δ*fur* deletion mutant strain after 48 h.

**Figure 6:**
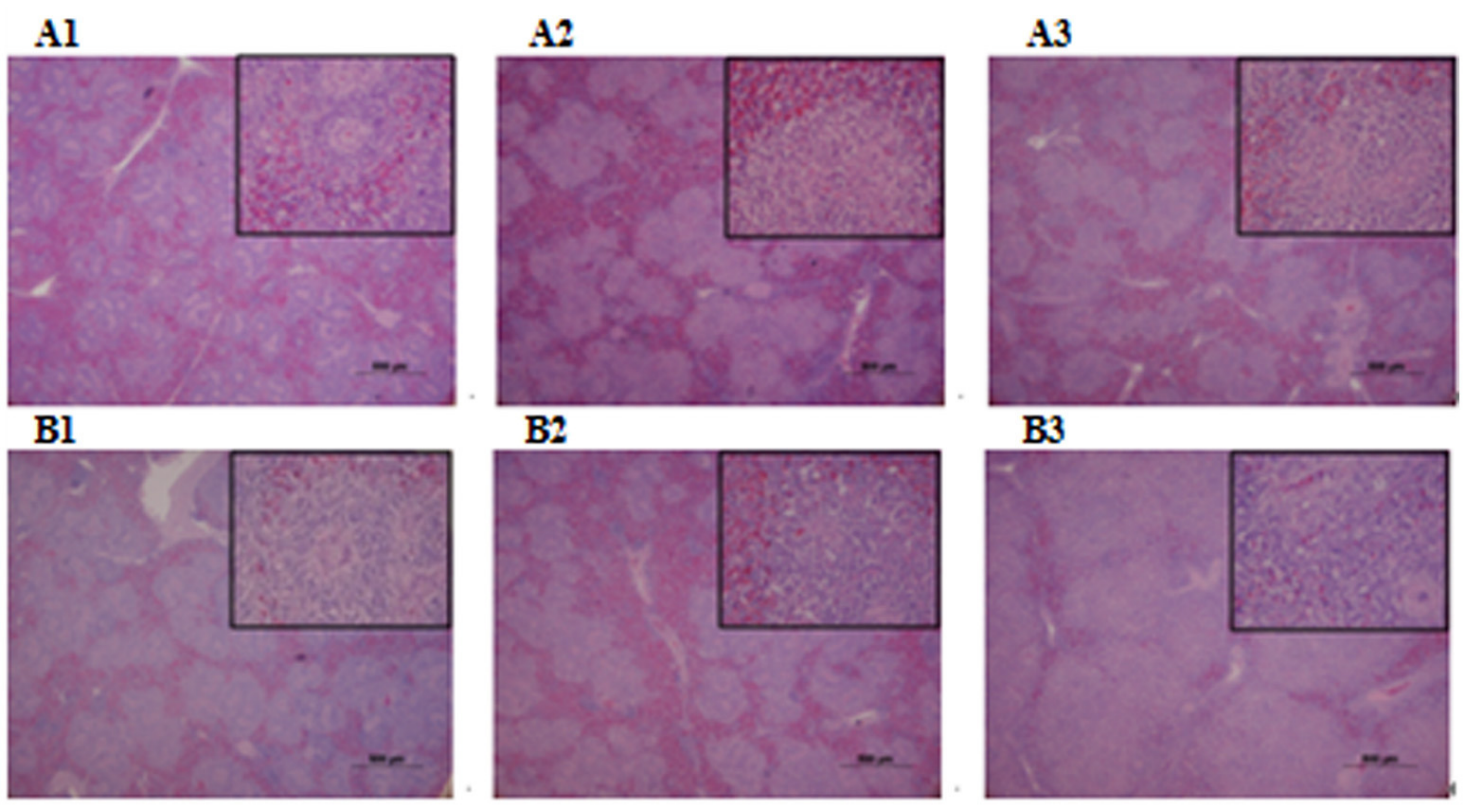
Histopathological diagram of spleen. **(A1)** The blank control group after 24 h, **(A2)** The group with wild type RA-YM strain after 24 h, **(A3)** The group with RA-YM Δ*fur* deletion mutant strain after 24 h. **(B1)** The blank control group after 48 h, **(B2)** The group with wild type RA-YM strain after 48 h, **(B3)** The group with RA-YM Δ*fur* deletion mutant strain after 48 h.

Microbiological analysis of heart, brain, liver and spleen showed that bacterial load was higher in wild type RA-YM strain compared to RA-YM Δ*fur* deletion mutant strain at 24 and 48 h post-infection collection, a detailed comparison is shown in Figures 2A,B. Correspondingly, the pathological investigations illustrated that lesions were more significant in wild type as compared to mutant strains. The epicardial tissue of ducklings infected with wild type bacteria consisted of a higher degree of fibrinous exudate and inflammatory cell infiltration as compared to infection with mutant pathogens after 24 and 48 h (Figure 3). The lesions in brain tissue after both 24 and 48 h, the subarachnoid space was examined where mild inflammatory cell infiltration was noted in case of RA-YM Δ*fur* deletion mutant strain as compared to wild type RA-YM strains (Figure 4). Similarly, a large number of hepatocytes expressed fatty degeneration and slight fibrotic effusion when infected with wild type RA-YM bacteria, whereas, such lesions were hardly observed in ducklings infected with RA-YM Δ*fur* mutant bacteria after 24 h inoculation. A higher degree of fibrotic effusions in liver tissue was observed after 48 h of infection with wild type pathogens, in comparison to mutant pathogens. Collectively, severe hepatic congestion was noticed in ducklings infected with wild type RA-YM strains (Figure 5). Likely, both splenomegaly and congestion of spleen was observed in ducklings after 24 and 48 h in case of infection with wild type pathogens, whereas only splenomegaly was noticed in case of infection with mutant pathogens (Figure 6). Conclusively, all groups of ducklings, inoculated with wild type RA-YM strains, were severely infected in comparison to those inoculated with RA-YM Δ*fur* deletion mutant strains. The control group of ducklings showed no significant pathological lesions.

**Figure 7:**
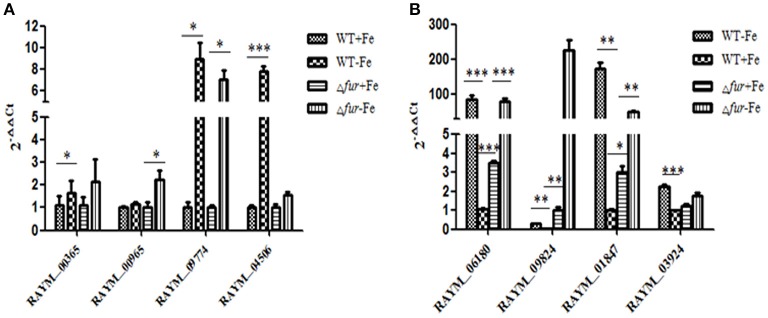
Real-Time PCR analysis of the Fur-and Iron- regulated genes. **(A)** The genes (*RAYM_00365, RAYM_00965, RAYM_09774, RAYM_04506*) regulated by iron. **(B)** The genes (*RAYM_06180, RAYM_09824, RAYM_01847, RAYM_03924*) regulated by Fur.

**Figure 8:**
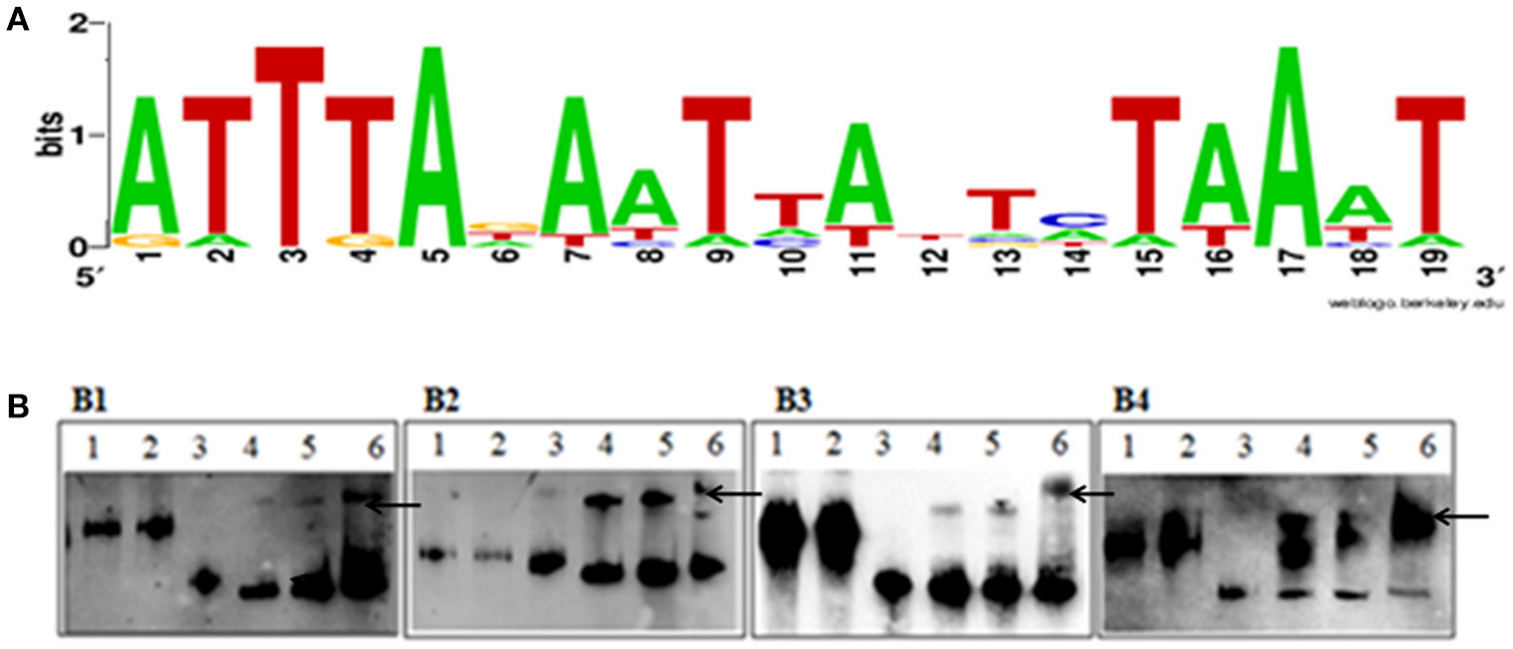
**(A)** Sequence logo of the Fur box of RA-YM. The binding sequence was listed by using WEBLOGO program. **(B)** EMSA of the Fur protein and its putative target promoters. **(B1)** Lane 1, DNA fragment of 16S rRNA; Lane 2, DNA fragment of 16S rRNA and Fur protein; Lane 3, DNA fragment of *RAYM_01847*; Lane 4, DNA fragment of *RAYM_01847* and Fur protein (0.1 μg); Lane 5, DNA fragment of *RAYM_01847* and Fur protein (1 μg); Lane 6, DNA fragment of *RAYM_01847* and Fur protein (10 μg); **(B2)** Lane 1, DNA fragment of 16S rRNA; Lane 2, 1 DNA fragment of 16s rRNA and Fur protein; Lane 3, DNA fragment of *RAYM_03924*; Lane 4, DNA fragment of *RAYM_03924* and Fur protein (0.1 μg); Lane 5, DNA fragment of *RAYM_03924* and Fur protein (1 μg); Lane 6, DNA fragment of *RAYM_03924* and Fur protein (10 μg); **(B3)** Lane 1, DNA fragment of 16S rRNA; Lane 2, DNA fragment of 16S rRNA and Fur protein; Lane 3, DNA fragment of *RAYM_06180*; Lane 4, DNA fragment of *RAYM_06180* and Fur protein (0.1 μg); Lane 5, DNA fragment of *RAYM_06180* and Fur protein (1 μg); Lane 6, DNA fragment of *RAYM_06180* and Fur protein (10 μg); **(B4)** Lane 1, DNA fragment of 16S rRNA; Lane 2, DNA fragment of 16S rRNA and Fur protein; Lane 3, DNA fragment of *RAYM_09824*; Lane 4, DNA fragment of *RAYM_09824* and Fur protein (0.1 μg); Lane 5, DNA fragment of *RAYM_09824* and Fur protein (1 μg); Lane 6, DNA fragment of *RAYM_09824* and Fur protein (10 μg); each lane the concentration of DNA fragment was 1 μg. The band which marked by arrows shows the Fur protein and DNA fragment complex.

## Discussion

In the present study, we employed the suicide vector pRE-lacZ-mpheS-spc-fur to construct an unmarked mutant RA-YM Δ*fur* successfully. Using this technique, the traditional method of mutant development by inducing antibiotic resistance can be circumvented, and influence on the expression of downstream genes can be minimized in some cases. However, the expression of the *fur* downstream gene *RAYM_04841* remained unchanged. The counter-selectable marker *pheS* in combination with *lacZ* have been successfully developed for unmarked gene deletion in RA. Previously, *pheS* had been also successfully used in *Burkholderia* (Barrett et al., 2008) and *E. faecalis* (Kristich et al., 2007), and *S. mutans* (Xie et al., 2011). The technique was developed to determine the role of *fur* in the pathogenicity of RA. Indeed, the virulence of RA-YM Δ*fur* mutant strain was attenuated in comparison to wild type and virulence was partially restored when RA-YM Δ*fur* mutant strain was complemented with the plasmid pRES-JXrep-spc. Moreover, bacterial load in different tissues was significantly decreased in RA-YM Δ*fur* mutant infection as compared to wild type strain. Similarly significantly mild lesions were observed in case of RA-YM Δ*fur* mutant infection in comparison to wild type infection. Conclusively, it was observed that Fur regulated virulence factors of RA infection. Indeed, the role of Fur with respect to virulence has been previously examined in *Vibrio cholerae* (Mey et al., 2005) and *Staphylococcus aureus* (Johnson et al., 2011). Consequently, findings of this study depicted the role of *fur* in virulence of RA.

In the current study, we also recorded the expression of the genes downregulated by iron under iron-restricted conditions. Among those genes, six downregulated genes were involved in regulation of tricarboxylic acid (TCA) cycle, which play an important role in metabolism, energy generation and synthesis of precursors (Vuoristo et al., 2016). Under iron-restricted conditions, certain key enzymes of the TCA cycle of RA-YM Δ*fur* strain were downregulated, which included succinate dehydrogenase (SDH) subunit (*RAYM_01977, RAYM_01982, RAYM_01987*), fumarate hydratase (*RAYM_00925*) and aconitase. SDH is involved in the respiratory chain and Krebs cycle of bacteria (Yankovskaya et al., 2003). Similarly, glyoxylate bypass pathways are also repressed in *Yersinia pestis* iron-restricted conditions (Pieper et al., 2010). In previous reports, downregulation of SDH, fumurase and aconitase were reported in *E. coli* (Massé et al., 2005) and *Bacillus subtilis* (Gaballa et al., 2008) in iron-sparing conditions. This phenomenon may be accounted for as the bacteria utilize an alternative iron-independent pathway of the TCA cycle and repressed numerous of iron-containing proteins under iron–restricted conditions. Moreover, cysteine synthase A (*cysK*, a significant enzyme of cysteine biosynthesis) and serine O-acetyltransferase (*cysE*, catalyzes the acetylation of L-serine to O-acetyl-L-serine) involved in amino biosynthesis were also downregulated due to iron deficiency. This inhibition regulates the conversion of available serine to siderophore, enterobactin, which thereby increases iron acquisition (Salvail et al., 2010). The cytochrome c oxidase (Cco) family related to oxidation-reduction was also inhibited in response to iron limitation, which is comprised of four subunits, CcoN, CcoO, CcoP, and CcoQ, which act as the terminal enzyme of respiratory chain (Ahn et al., 2015; Steininger et al., 2016). The Cco family, a member of heme-copper oxidase superfamily, may play a role in iron- restricted conditions. In *Pseudomonas stutzeri*, the Cco family has also been reported as an essential element for nitrogen-fixing (Nyquist et al., 2001; Xie et al., 2014). Our data showed that nitrogen-fixing associated genes (*RAYM_07584, RAYM_07589*) were repressed in iron-restricted conditions. In conclusion, iron-sparing responses, which means the repression of iron-dependent genes when iron is deficient, was the vital reason of the genes downregulated by iron in iron restricted conditions.

Among genes induced by iron, the genes (*RAYM_00510, RAYM_00515*) associated with iron acquisition were upregulated, which included Ferrous iron transport protein A (FeoA) and Ferrous iron transport protein B (FeoB). The *feoB* gene encodes an inner membrane Fe (II) transporter in multiple bacteria, such as *E. coli* and *V. cholerae* whereas *feoA* gene was demonstrated crucial for FeoB uptake of Fe(II) (Marlovits et al., 2002; Kim et al., 2012). Other genes involved in the iron-acquisition system were also upregulated, including Ferritin (*RAYM_01160*), ABC transporter related protein (*RAYM_06607*), TonB-dependent receptor (*RAYM_04481*). This is an apparent response to iron starvation in bacteria, which has also been demonstrated in *Klebsiella pneumonia* and *Listeria monocytogenes* (Ledala et al., 2010; Lin et al., 2011). Similarly, a group of genes contributed to the biosynthetic process of iron-sulfur (Fe-S) were also regulated by iron. In bacteria, Fe-S machinery is comprised of a nitrogen-fixing NifU domain protein (*RAYM_01100*), SUF system protein (*RAYM_01495, RAYM_06457, RAYM_06467, RAYM_06507*). Similar changes of NIF and SUF systems have been confirmed in *E. coli* in previous studies (Outten et al., 2004). Moreover, the genes Phosphoserine aminotransferase (*RAYM_04219*), D-3-phosphoglycerate-dehydrogenase (*RAYM_04224*) controlling the shikimate pathway were upregulated. D-3-phosphoglycerate-dehydrogenase (pabB) converts chorismate to 4-amino-4-deoxychorismate (ADC) and phosphoserine aminotransferase (pabC) converts ADC to p-aminobenzoate (PABA) and pyruvate (Green et al., 1992). Shikimate pathway catalyzes serine to form siderophore, enterobactin (Prévost et al., 2007). The upregulation of the pabB and pabC in iron-limited conditions results in high levels of both aromatic amino acids and phenolate siderophore (Lemaître et al., 2014). Moreover, in our data, sigma factor protein (*RAYM _00365*) was upregulated by iron. Iron-starvation sigmas, a extracytoplamic function (ECFs) subfamily, have been demonstrated previously to play a role in iron acquisition in *P. aeruginosa* (Visca et al., 2002). In conclusion, the genes induced by iron are involved in iron-acquisition, some metabolic pathways and several transcriptional regulation factors. The series of regulatory responses under low-iron conditions resulted in increasing iron acquisition.

Among the genes regulated by Fur under iron-restricted conditions, Hmu system, comprised of *hmuY* (*RAYM_06175*) and *hmuR* (*RAYM_06180*), was observed to be regulated by Fur. The homologous Fur-box sequence has been identified upstream of the *hmuY* start codon in *Porphyromonas gingivalis* (Simpson et al., 2000). HmuR, the TonB-dependent receptor for ferric, has previously been illustrated to be regulated by Fur in *Y. pestis* (Branger et al., 2010). Furthermore, genes (*RAYM_01847, RAYM_09824*, and *RAYM_09779*) encoding TonB-dependent outer membrane proteins are also regulated by Fur under iron-restricted conditions. The accessory proteins of TonB system can transduce energy in Gram-negative bacteria (Postle and Kadner, 2003; Lim et al., 2012). Our data confirmed that TonB-ExbB-ExbD system was a significant component for ferric enterobactin acquisition, which was previously reported in *Campylobacter* (Zeng et al., 2013). In addition, *RAYM_00450* (oxidoreductase), *RAYM_03864* (3-hydroxyacyl-CoA dehydrogenase) were also regulated by Fur which was demonstrated in previous study as Fur regulated the response to oxidative stress in *C. jejuni* (Holmes et al., 2005). Interestingly, *sprT* gene (*RAYM_03924*), a component of the type IX secretion system (T9SS) was also regulated by Fur. Parallel findings were recorded in *Flavobacterium johnsoniae* (Kharade and McBride, 2015). T9SS was also characterized as a novel protein secretion system mediated outer membrane translocation to the cell surface in *P. gingivalis* (de Diego et al., 2016). In conclusion, Fur regulated processes included iron acquisition, oxidation and reduction, and regulation of some components of T9SS.

Fur has been reported to act as a negative regulator. It was demonstrated that Fur protein, together with iron, can bind to a consensus sequence, resulting in transcriptional inhibition (Baichoo and Helmann, 2002) which had been described in *E. coli, P. aeruginosa, Helicobacter pylori*, and *H. hepaticus* (Escolar et al., 1998; Vasil and Ochsner, 1999; Belzer et al., 2007; Pich et al., 2012). The Fur-box consensus sequence 5′-GATAATGATAATCATTATC-3, was slightly different among bacteria. It was identified as an adjacent hexamer unit of the sequence 5′-GATTAT-3′ or three repeat of the NATWAT (Lavrrar and McIntosh, 2003). Typically, the Fur binding sequence was located within 150 bp of the translation initiation codon of the regulated genes (Grifantini et al., 2003). The putative Fur-box sequence of RA-YM in our study was predicted as 5′-ATTTAGAATTATTCTAAAT- 3′, and the sequences might be located within 100 bp of the translation initiation codon of regulated genes which could be reasoned due to the unique promoter of the strain. RA-YM belongs to the *Flavobacterium*, where promoter have −7 and −33 consensus elements, whereas the promotor of *E. coli* has −10 and −35 consensus elements (Chen et al., 2007).

In summary, our work showed that *pheS* acted as effective counter-selectable marker for conjugal transfer. We successfully constructed an unmarked deletion mutant of RA with the suicide vector pRE-lacZ-mpheS-spc. In addition, we elucidated the role of the *fur* gene in virulence of RA. Furthermore, we screened out the genes regulated by iron and Fur. The putative Fur-box sequence of RA was also predicted. Conclusively, this was a comprehensive study on the metabolism of *R. anatipestifer* which may help facilitate the control of this pathogen.

## Author contributions

YG designed, performed the experimentation, data analysis and wrote the manuscript. DH, JG, XL, and JYG performed experimentations. XW, YX, HJ, ML, and ZL designed and contributed to experimental work. ZZ and DB designed, analyzed the data and revised the manuscript. All authors read and approved the final manuscript.

### Conflict of interest statement

The authors declare that the research was conducted in the absence of any commercial or financial relationships that could be construed as a potential conflict of interest.
