# Supplementary material for: The Role of the Regulator Fur in Gene Regulation and Virulence of Riemerella anatipestifer Assessed Using an Unmarked Gene Deletion System
Source: Front Cell Infect Microbiol. 2017 Aug 25;7:382. doi: 10.3389/fcimb.2017.00382 (PMC5609570; doi:10.3389/fcimb.2017.00382)
Supplement: Supplementary file 1 [file Presentation1.PDF]

（A）

```
pheS    ATGTTAGAATACATTGACGCATATCTACAAGAGGTAAAGCAATTTCAGTCTTCAAACAAAGATGAAATAGAGCAATTCAG    80
mpheS   ATGTTAGAGTATATTGATGCTTACCTTCAAGAAGTTAAACAGTTCCAATCTAGCAATAAGGACGAAATTGAACAGTTTAG    80

pheS    AATAAAATTTAACGGTAAAAAAGGAATTTTAAATGCCATCTTTGATGAGTTTAAACAAGTTCCGAACGAACAAAAAAAGG    160
mpheS   AATTAAGTTCAATGGAAAAAAGGGTATTCTTAACGCTATTTTCGATGAATTTAAGCAAGTACCAAATGAGCAGAAAAAAG    160

pheS    CTTTTGGACAAAAAATAAATGTTCTAAAACAAGCTGTTGCCGAAAAACTAGAAGAGCTTAAAAATGCAACCGCCTCTAGT    240
mpheS   CATTCGGACAAAAGATCAATGTATTAAAGCAGGCAGTAGCTGAAAAATTAGAGGAATTAAAAAACGCTACTGCTTCATCT    240

pheS    ATTGTTGTAGAAAAAGAAGATTTAACTCGTCCTGGGTATCCTTTGGAATTGGGGAGCAGACACCCTATCAACTTAGTTAA    320
mpheS   ATTGTAGTTGAAAAGGAAGACCTTACAAGACCAGGATATCCATTAGAACTTGGATCAAGACATCCAATTAATCTTGTAAA    320

pheS    GAATAGAATTATCGAGATATTTAAGTCCATAGGGTTTGCCGTGTCAGACGGTCCAGAAATAGAGGACGACTGGCACAACT    400
mpheS   AAACAGAATTATTGAAATTTTTAAAAGCATAGGTTTCGCTGTATCTGATGGTCCTGAGATAGAAGATGATTGGCATAATT    400

pheS    TTACAGCCCTTAACCTCCCCGAATATCACCCTGCTAGAGATATGCAGGATACGTTTTTCATAGAGCAGAATCCTGACACC    480
mpheS   TCACTGCTTTGAATTTACCTGAGTATCATCCAGCAAGAGACATGCAAGATACATTTTTTATAGAACAAAACCCAGATACT    480

pheS    CTCCTTAGAACACACACTTCGTCTGTGCAGATAAGACATATGGAACAGAACCAACCTCCAATGCGTATTCTATCACCGGG    560
mpheS   CTTTTAAGAACTCATACATCTAGCGTACAAATTAGACACATGGAGCAAAATCAGCCACCTATGAGAATCCTTTCTCCTGG    560

pheS    TAGAGTATTTAGAAATGAGGCTATTTCTTCTCGTTCGCATTGTATTTTCCACCAGATAGAAGGACTTTATATTGATGAAA    640
mpheS   AAGAGTTTTTAGAAACGAAGCAATTTCTTCAAGATCTCACTGTATCTTTCATCAAATTGAGGGATTGTACATTGACGAAA    640

pheS    AGGTAAGTTTTGCGGATTTGAAGCAAACCATACAGTTTTTCACTACGGAGCTTTTTGGAAAGTCTAAAATTAGAATGAGA    720
mpheS   AAGTTTCATTCGCAGATCTTAAACAGACTATACAATTTTTTACAACAGAATTATTCGGTAAATCAAAGATCAGAATGAGA    720

pheS    CCGTCTTATTTCCCATTCACCGAGCCAAGTGCGGAGGTTGATGTTTATTGGGGGCTTAACTCCGAAACAGACTACCGAAT    800
mpheS   CCTTCATATTTTCCTTTTACAGAACCTTCAGCTGAAGTAGACGTATACTGGGGTTTAAATTCTGAAACTGATTATAGAAT    800

pheS    TACTAAAGGTACAGGTTGGTTAGAAATTATGGGCTGTGGTATGGTAGACCCTGCCGTACTTAAAAATGTAAATATAGACC    880
mpheS   TACAAAGGGCACTGGTTGGCTTGAGATCATGGGTTGTGGAATGGTTGATCCAGCTGTTTTAAAGAACGTTAACATAGATC    880

pheS    CTGACAAATACAGTGGCTATGCTTTTGGTATGGGAATAGAGCGAATTGTAATGCTTCTCTTCCAAATGAGCGACATTCGT    960
mpheS   CAGATAAGTATTCAGGTTACGGCTTCGGAATGGGTATTGAAAGAATTGTTATGTTACTTTTTCAGATGTCAGATATTAGA    960

pheS    ATGTTCTTTGAGAACGATGTAAGAATGCTAGAACAGTTTAAAACGCTATAA                              1011
mpheS   ATGTTTTTCGAAAATGACGTTCGTATGTTAGAGCAATTCAAAACATTATAA                              1011
```

（B）

```
pheS    MLEYIDAYLQEVKQFQSSNKDEIEQFRIKFNGKKGILNAIFDEFKQVPNEQKKAFGQKINVLKQAVAEKLEELKNATASS    80
mpheS   MLEYIDAYLQEVKQFQSSNKDEIEQFRIKFNGKKGILNAIFDEFKQVPNEQKKAFGQKINVLKQAVAEKLEELKNATASS    80

pheS    IVVEKEDLTRPGYPLELGSRHPINLVKNRIIEIFKSIGFAVSDGPEIEDDWHNFTALNLPEYHPARDMQDTFFIEQNPDT    160
mpheS   IVVEKEDLTRPGYPLELGSRHPINLVKNRIIEIFKSIGFAVSDGPEIEDDWHNFTALNLPEYHPARDMQDTFFIEQNPDT    160

pheS    LLRTHTSSVQIRHMEQNQPPMRILSPGRVFRNEAISSRSHCIFHQIEGLYIDEKVSFADLKQTIQFFTTELFGKSKIRMR    240
mpheS   LLRTHTSSVQIRHMEQNQPPMRILSPGRVFRNEAISSRSHCIFHQIEGLYIDEKVSFADLKQTIQFFTTELFGKSKIRMR    240

pheS    PSYFPFTEPSAEVDVYWGLNSETDYRITKGTGWLEIMGCGMVDPAVLKNVNIDPDKYSGYAFGMGIERIVMLLFQMSDIR    320
mpheS   PSYFPFTEPSAEVDVYWGLNSETDYRITKGTGWLEIMGCGMVDPAVLKNVNIDPDKYSGYGFGMGIERIVMLLFQMSDIR    320

pheS    MFFENDVRMLEQFKTL                                                                  336
mpheS   MFFENDVRMLEQFKTL                                                                  336
```

FIG 1 (A) The mutant *pheS* gene was engineered by substituting alternative bases at numerous positions to reduce the probability of undesired homologous recombination at the locus. (B) The mutant PheS protein was highly conserved compared to the *Riemerella anatipestifer* PheS.
